# Manganese-Enhanced Magnetic Resonance Imaging in Takotsubo Syndrome

**DOI:** 10.1161/CIRCULATIONAHA.122.060375

**Published:** 2022-11-01

**Authors:** Singh T, Joshi S, Kershaw LE, Baker AH, McCann GP, Dawson DK, Dweck MR, Semple SI, Newby DE

**Affiliations:** 1 BHF/University Centre for Cardiovascular Science, University of Edinburgh, UK; 2 Edinburgh Heart Centre, Royal Infirmary of Edinburgh, UK; 3 Edinburgh Imaging, University of Edinburgh, UK; 4 Aberdeen Cardiovascular and Diabetes Centre, University of Aberdeen, Aberdeen, UK; 5 Department of Cardiovascular Sciences, University of Leicester and NIHR Leicester Biomedical Research Centre, Glenfield Hospital, UK

**Keywords:** Takotsubo syndrome, Manganese-enhanced magnetic resonance imaging, Myocardial calcium handling

## Abstract

**Background:**

Takotsubo syndrome is an acute cardiac emergency characterized by transient left ventricular systolic dysfunction typically following a stressful event. Despite its rapidly rising incidence, its pathophysiology remains poorly understood. Furthermore, it may pass unrecognized especially if timely diagnostic imaging is not performed. Defective myocardial calcium homeostasis is a central cause of contractile dysfunction and has not been explored in takotsubo syndrome. We aimed to investigate myocardial calcium handling using manganese-enhanced magnetic resonance imaging during the acute and recovery phases of takotsubo syndrome.

**Methods:**

Twenty patients with takotsubo syndrome (63 ± 12 years, 90% female) and 20 age, sex and cardiovascular risk factor matched volunteers (59 ± 11 years, 70% female) were recruited from the Edinburgh Heart Centre between March 2020 and October 2021. Patients underwent gadolinium and manganese-enhanced magnetic resonance imaging during index hospitalization with repeat manganese-enhanced magnetic resonance imaging performed after at least 3 months.

**Results:**

Compared to matched control volunteers, patients had a reduced left ventricular ejection fraction (51±11 versus 67±8 %, P<0.001), increased left ventricular mass (86±11 versus 57±14 g/m^2^, P<0.001) and, in affected myocardial segments, elevated native T1 (1358±49 versus 1211±28 ms, P<0.001) and T2 (60±7 versus 38±3 ms, P<0.0001) values at their index presentation. During manganese-enhanced imaging, kinetic modelling demonstrated a substantial reduction in myocardial manganese uptake (5.1±0.5 versus 8.2±1.1 mL/100 g of tissue/min respectively, P<0.0001) consistent with markedly abnormal myocardial calcium handling. Following recovery, left ejection fraction, left ventricular mass and T2 values were comparable to matched control volunteers. Despite this, native and post-manganese T1 and myocardial manganese uptake remained abnormal compared to matched control volunteers (6.6±0.5 versus 8.2±1.1 mL/100 g of tissue/min, P<0.0001).

**Conclusions:**

In patients with takotsubo syndrome, there is a profound perturbation of myocardial manganese uptake which is most marked in the acute phase but persists for at least 3 months despite apparent restoration of normal left ventricular ejection fraction and resolution of myocardial edema, suggesting abnormal myocardial calcium handling may be implicated in the pathophysiology of takotsubo syndrome. Manganese-enhanced magnetic resonance imaging has major potential to assist in the diagnosis, characterization, and risk stratification of patients with takotsubo syndrome.

## Introduction

Takotsubo syndrome is an acute cardiac emergency that is often triggered by a stressful event and is characterized by transient and profound left ventricular systolic dysfunction, typically due to marked ‘ballooning’ of the left ventricular apex.^1–3^ The pathology of this condition is poorly understood and we lack targeted treatments. Moreover, it can be challenging to recognize due to its phenotypical similarities with acute myocardial infarction and is often considered when invasive coronary angiography fails to identify major obstructive coronary artery disease.^4^ However, the characteristic left ventricular abnormalities can be brief, and opportunities to document them with diagnostic imaging are often missed.^5^

Cardiac magnetic resonance imaging is an essential diagnostic tool in the assessment of takotsubo syndrome. In addition to visualizing the hallmark regional wall motion abnormalities, it can identify complications, such as left ventricular outflow tract obstruction, mitral regurgitation, pericardial effusion and left ventricular thrombus. It is particularly useful in establishing the diagnosis of acute cardiac conditions of uncertain origin, such as myocardial infarction with non-obstructive coronary arteries and myocarditis. Previous cardiac magnetic resonance imaging studies have demonstrated the presence of acute myocardial edema in patients with takotsubo syndrome as well as some reports of persistent subtle cardiac abnormalities following recovery of normal left ventricular ejection fraction.^6^ However, there is a need to develop more sensitive and discriminatory imaging techniques that are less time sensitive and more specific to takotsubo syndrome.

Manganese, a calcium ion analogue, has paramagnetic properties and can cross intact cell membranes via voltage-gated calcium channels, providing intracellular contrast of viable tissue on magnetic resonance imaging. In 1981, Hunter et al proposed that uptake of free manganese ions in the myocardium can be used to measure calcium uptake because manganese is retained intracellularly.^7^ Manganese-enhanced magnetic resonance imaging thus represents a promising novel approach to the assessment of myocardial calcium handling. We have previously demonstrated differences in manganese uptake in patients with dilated, hypertrophic or ischemic cardiomyopathy, suggesting abnormal myocardial calcium handling.^8, 9^ However, there have been no studies assessing whether manganese-enhanced magnetic resonance imaging can detect alterations in myocardial calcium handling in takotsubo syndrome and whether this recovers during convalescence. The objectives of this proof-of-concept study were to evaluate the ability of manganese-enhanced magnetic resonance imaging to detect altered myocardial calcium handling in patients with takotsubo syndrome during both the acute presentation and following apparent recovery.

## Methods

This was a single center case-control observational longitudinal cohort study (NCT04623788) which was conducted in accordance with the Declaration of Helsinki, the favourable ethical opinion of the Southeast Scotland Research Ethics Committee 2 (20/SS/0001) and with the written informed consent from all participants. The data, analytic methods, and study materials will be made available to other researchers on reasonable request.

### Study Populations

Adult patients (≥18 years of age) with takotsubo syndrome were recruited from the Edinburgh Heart Centre between March 2020 and October 2021. Diagnosis of takotsubo syndrome was based on the Mayo clinic^10^ and the Heart Failure Association Takotsubo Syndrome Taskforce of the European Society of Cardiology criteria.^11^ This comprises of new electrocardiographic (ECG) changes (ST-segment elevation, ST-segment depression, T-wave inversion, and QTc prolongation), the presence of transient left ventricular dysfunction presenting as apical ballooning or focal mid-ventricular/basal wall motion abnormalities, and the absence of obstructive coronary artery disease or acute plaque rupture. Patients usually have an emotional or physical stressful trigger. We specifically excluded patients with pheochromocytoma, myocarditis or a primary isolated diagnosis of acute myocardial infarction.

Control volunteers were selected consecutively and were matched to patients with takotsubo syndrome 1:1, for age, sex and cardiovascular risk factor profile, such as hypertension, known coronary artery disease, hypercholesterolemia and diabetes mellitus. All participants were scanned at the University of Edinburgh between September 2020 and August 2021.

Exclusion criteria for all participants were any contraindication to magnetic resonance imaging, contraindications to manganese dipyridoxyl diphosphate administration (high degree atrioventricular block, history of torsades de pointes or prolonged QTc interval, obstructive liver disease), ongoing calcium channel antagonist or digoxin therapy, renal failure (estimated glomerular filtration rate <30 mL/min/1.73 m^2^), New York Heart Association class IV heart failure, and women of child-bearing potential without a negative pregnancy test. Subjects receiving a calcium channel antagonist had their medication withheld 48 hours prior to manganese-enhanced imaging.

#### Magnetic Resonance Imaging

**Figure 1:**
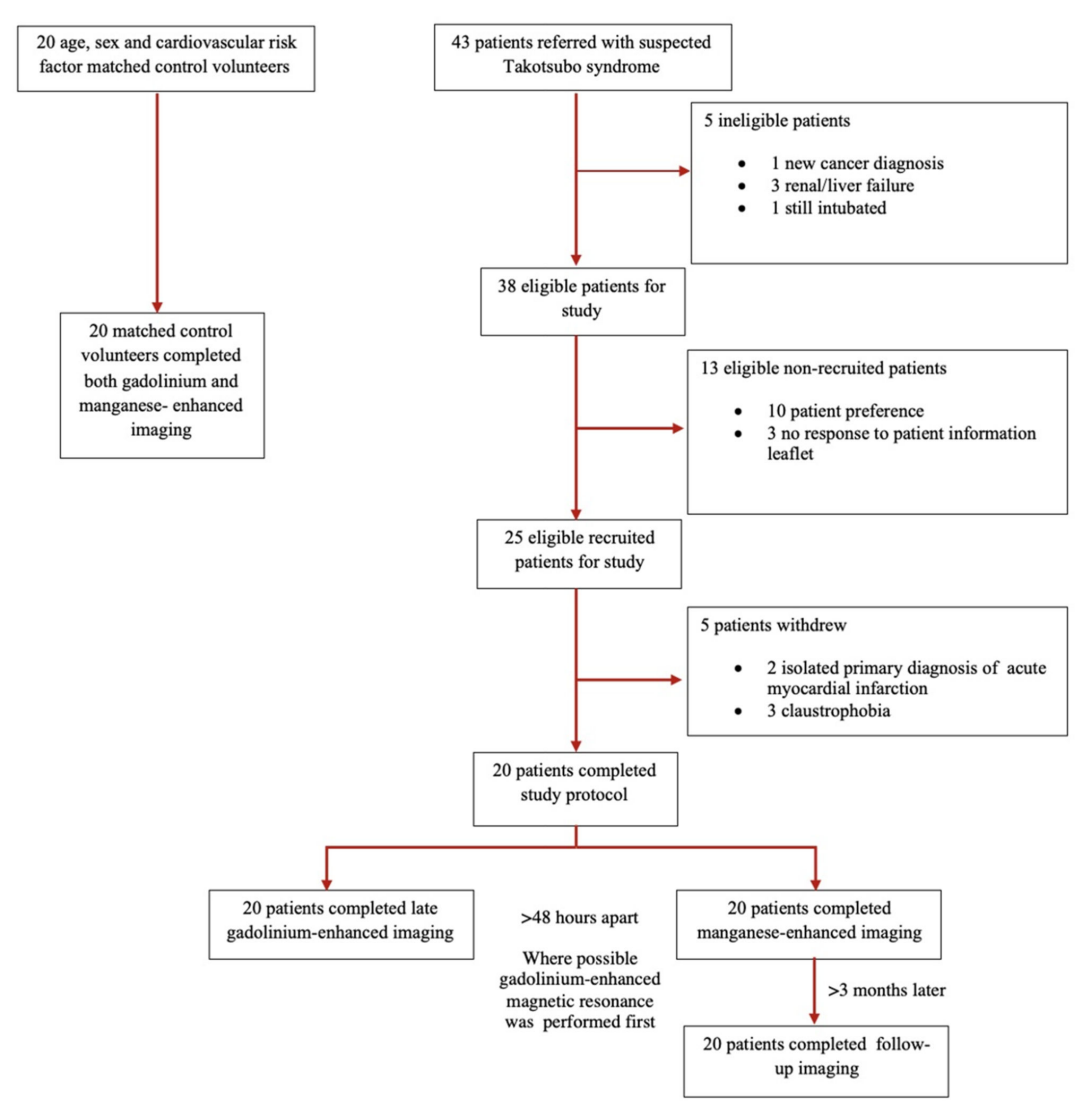
Consort Diagram

Magnetic resonance imaging was performed using a 3T scanner (MAGNETOM Skyrafit, Siemens Healthineers, Erlangen, Germany) using a 30-channel anterior body matrix coil and elements of a posterior spine matrix coil. Images were acquired during expiratory breath hold with ECG gating. Cine imaging was acquired with standard steady-state free precession sequences in long and short-axis orientations as described previously.^8, 9, 12^ All study participants underwent scanning with both late-gadolinium enhancement and manganese-enhanced magnetic resonance imaging, at least 48 hours apart. Where possible, gadolinium imaging was performed first, with at least 48 hours between the two scans (Figure 1).

T1 mapping was performed prospectively with modified Look-Locker inversion (MyoMaps) recovery (repetition time 388.8 ms; echo time 1.07 ms; matrix 256×115; slice thickness 8 mm with 1.6-mm gap, FOV=360×288 mm, sampling pattern 5(3)3). Quantitative estimation of T1 was performed in full short-axis stack from mitral valve annulus to apex and standard long-axis slices, with additional slices positioned appropriately to characterize pathology.

T2 mapping was performed with T2 Myomaps (TR 207.39 ms; TE 1.32 ms; matrix 192×100; slice thickness 8 mm with 1.6 mm gap, with T2 evolution times of 0, 0.30 and 0.55 ms. Field of view (FOV) was 360×288 mm adjusted for patient body habitus as required (MyoMaps, Siemens Healthineers, Erlangen, Germany). This was acquired in long and short-axis orientation covering the entire left ventricle.

##### Late-Gadolinium Enhancement

Late-gadolinium enhancement images were acquired following intravenous administration of gadobutrol (0.1 mmol/kg; Gadovist, Bayer, Germany) using a single breath held phase-sensitive inversion recovery short-axis stack, and long axis orientations (TR 820 ms; TE 1.04 ms; matrix 192×81; slice thickness 8 mm with 1.8-mm gap, FOV 380 mm). A standardized inversion time of 400 ms was used and adjusted as required for optimal myocardial nulling. Post-contrast T1 mapping was performed prospectively with short-axis modified Look-Locker inversion recovery stack 10 min after contrast administration.

##### Manganese-enhanced magnetic resonance imaging

Manganese-enhanced magnetic resonance imaging was achieved using intravenous infusion of manganese dipyridoxyl diphosphate (5 μmol/kg, 0.1 mL/kg, up to a maximum of 10 mL, 1mL/min; Exova SL Pharma, Wilmington, Delaware, USA) T1 mapping was performed pre-contrast with full short-axis modified Look-Locker inversion recovery stack as above. For patients, a single short-axis slice of the diseased myocardium was identified by the supervising cardiologist and guided by native T1, T2 maps and cine images (mid-ventricular or basal in all patients). Single short-axis T1 mapping was then performed at this slice location every 2.5 min for 30 min after commencing contrast infusion. At 30 min, a full short-axis T1 stack was repeated (Figure S1). For controls, a mid-ventricular slice was chosen for serial T1 mapping after manganese dipyridoxyl diphosphate infusion.

#### Image Analysis

All analyses of T1, T2 maps, late-gadolinium enhancement and cine-derived volumetric and functional sequences were performed using Circle CVI (Circle Cardiovascular Imaging, CVI42 v5.3.6, Calgary Canada) as previously validated and described in animal and human models.^8, 9, 13^ Image analysis was conducted blind to patient’s details (analysed in groups after the end of scanning period) and manganese-enhanced images were analysed separately from late-gadolinium images. Endocardial and epicardial borders were manually defined on all conventional short-axis images for volumetric and wall motion measurements and were then copied to corresponding T1 map images for analysis with minimal manual adjustments. The left ventricular basal short axis slice was identified as the image containing at least 50% of circumferential myocardium at end diastole. Papillary muscles were included in the mass and excluded from volumetric analysis.

After contouring, an additional epicardial and endocardial offset of 20% was applied automatically to minimize partial volume effect for all T1 map analyses. In patients, T1 and T2 measurements were taken from septal segments in both pathological (area with regional wall motion abnormality) and remote regions (no regional wall motion abnormality). In control volunteers, T1 and T2 measurements were made in the septal wall of the midventricular slice (Figure S2). Global native, post-manganese T1 and Ki (mean of 6 segments from a short-axis slice) were also measured in patients (excluding focal takotsubo and dual pathology, n=4) and volunteers (Table S1). Left ventricular wall thickness was measured in the pathological and remote myocardium (septal walls for both) for acute and follow-up scans. For matched volunteers, wall thickness was measured in the mid-ventricular septal wall (Figure S3).

#### Manganese Kinetic Modelling

Manganese uptake was calculated for a selected single short-axis slice of the diseased myocardium which was chosen based on the presence of regional wall motion abnormality, and native T1 and T2 maps. To quantify the change in T1 over time, regions of interest were drawn as described above. For serial T1 imaging post manganese, manually drawn regions of interest from the pre-contrast image were transferred to all subsequent post-contrast images to ensure consistency. The rate of myocardial manganese uptake (Ki) was determined by Patlak modelling as described previously.^12, 14, 15^

#### Statistical analysis

All statistical analysis was performed with GraphPad Prism (GraphPad Software v8.0.2, San Diego, California, USA). Categorical baseline variables were presented as number (%) and compared using Chi-squared test. Continuous data were assessed for normality using the D’Agostino-Pearson test and presented as mean±standard deviation or median [interquartile interval]. Cardiac function, myocardial manganese uptake, volumetric assessment and parametric mapping values were compared using paired Student’s *t*-tests and Wilcoxon signed rank and unpaired t-test or Mann-Whitney tests where appropriate. Sensitivity analyses were performed excluding the focal subtype of takotsubo syndrome or the presence of dual pathology. Correlations were assessed using linear regression analysis. Statistical significance was taken as two-sided p<0.05.

## Results

### Study Populations

Twenty-five patients with takotsubo syndrome were recruited into the study although five patients were withdrawn: 3 were unable to complete the cardiac magnetic resonance scan due to claustrophobia and 2 had an infarct pattern of late-gadolinium enhancement on magnetic resonance imaging and an isolated primary diagnosis of acute myocardial infarction (Figure 1). The patient cohort was predominantly middle-aged women (Table 1). All patients had symptoms (chest pain in 70% and dyspnea in 30%) requiring emergency hospitalization with the majority having an identifiable precipitating stressor (Table S2). Only 2 patients had pre-existing symptoms (1 chest pain, 1 palpitation). Twenty control volunteers were well matched for age and co-morbidities although there was a higher prevalence of women and pre-existing psychiatric or neurological disorders in the patient cohort (Table 1). However, there were no demonstrable differences in myocardial manganese uptake according to the use of anti-depressant therapies (Table S3). Similarly, we found no difference in myocardial manganese uptake in our control cohort between women and men (8.2±0.7 versus, 8.3±1.0 mL/100 g of tissue/min, P=0.71). Five patients and one control volunteer were on prior calcium channel antagonist. All patients had this therapy discontinued at presentation due to the presence of left ventricular dysfunction and the control subject had their calcium channel antagonist withheld 48 hours before manganese-enhanced magnetic resonance imaging.

### Manganese Infusion

Sixty infusions of manganese dipyridoxyl diphosphate were completed during the course of the study (mean duration of 10 min). There were no changes in the electrocardiogram, heart rate or blood pressure (Figures S4) following manganese administration (P>0.1 for all). One control volunteer experienced mild transient nausea for <10 s after commencing manganese infusion, spontaneously resolving without intervention. Otherwise, contrast administration was well tolerated with no adverse reactions reported during or immediately after administration or at follow up at seven days.

### Characterization of Takotsubo Syndrome

**Figure 2:**
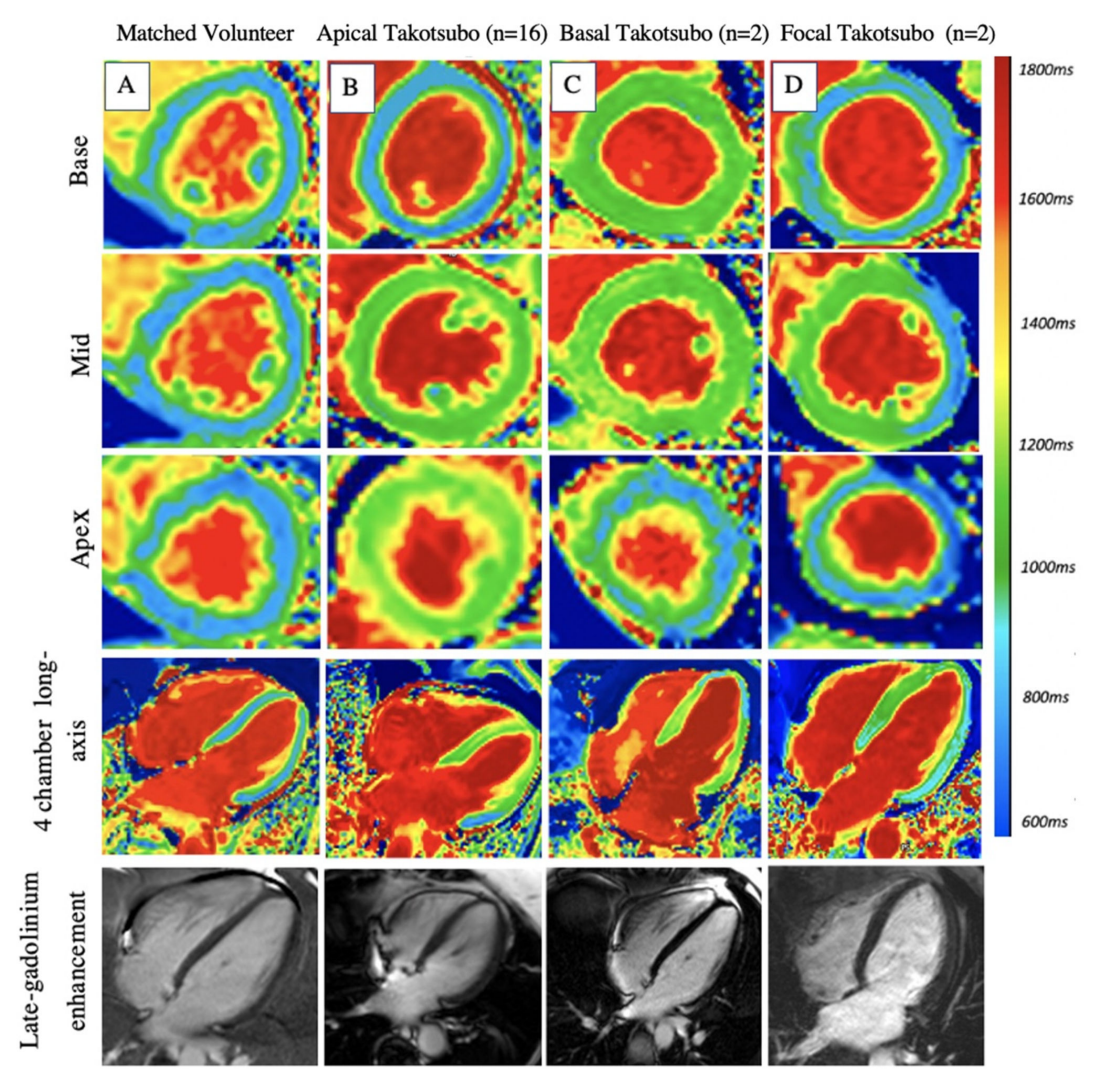
Anatomical Types of Takotsubo Syndrome Short-axis and long-axis manganese-enhanced T1 maps and long-axis late-gadolinium images (10 min post contrast) in a matched control volunteer (panel A), and patients with apical (panel B), basal (panel C) and focal (panel D) takotsubo syndrome. Blue represents normal manganese uptake and green represents reduced manganese uptake and abnormal calcium handling.

Electrocardiography demonstrated either ST-segment deviation or T-wave changes in all patients, with evidence of QT interval prolongation in some patients. All patients had a degree of left ventricular impairment on baseline echocardiographic imaging. All patients underwent invasive coronary angiography, which, demonstrated normal coronary arteries in half of the population, with the remaining having non-obstructive coronary artery disease (Table 1, Table S4). Two patients had evidence of acute coronary pathology: one had spontaneous coronary artery dissection in the first obtuse marginal and the second had plaque rupture in the distal left anterior descending artery. In both cases, coronary flow had been restored and there were extensive regional wall motion abnormalities involving all mid-ventricular and apical segments which were not in keeping with myocardial infarction alone. Most patients had an ‘apical’ pattern of takotsubo syndrome (Figure 2). Two patients demonstrated a ‘focal’ pattern of takotsubo: one of whom had normal coronary arteries whilst the other had mild plaque in the left anterior descending artery and underwent intra-coronary imaging (demonstrated stable plaque) and a pressure wire study (fractional flow reserve during maximal hyperemia, 0.92). Both patients demonstrated rapidly resolving left ventricular function and had no evidence of late-gadolinium enhancement. On discharge, all patients were commenced on heart failure treatment, with a smaller proportion being started on diuretic therapy. Patients who had evidence of coronary artery disease were commenced on anti-platelet therapy, and two patients were started on dual anti-platelet therapy (Table 1). During follow up, one patient suffered a stroke and another from recurrence of takotsubo syndrome within a year of their index event.

### Magnetic Resonance Imaging

#### Acute Index Presentation

**Figure 3:**
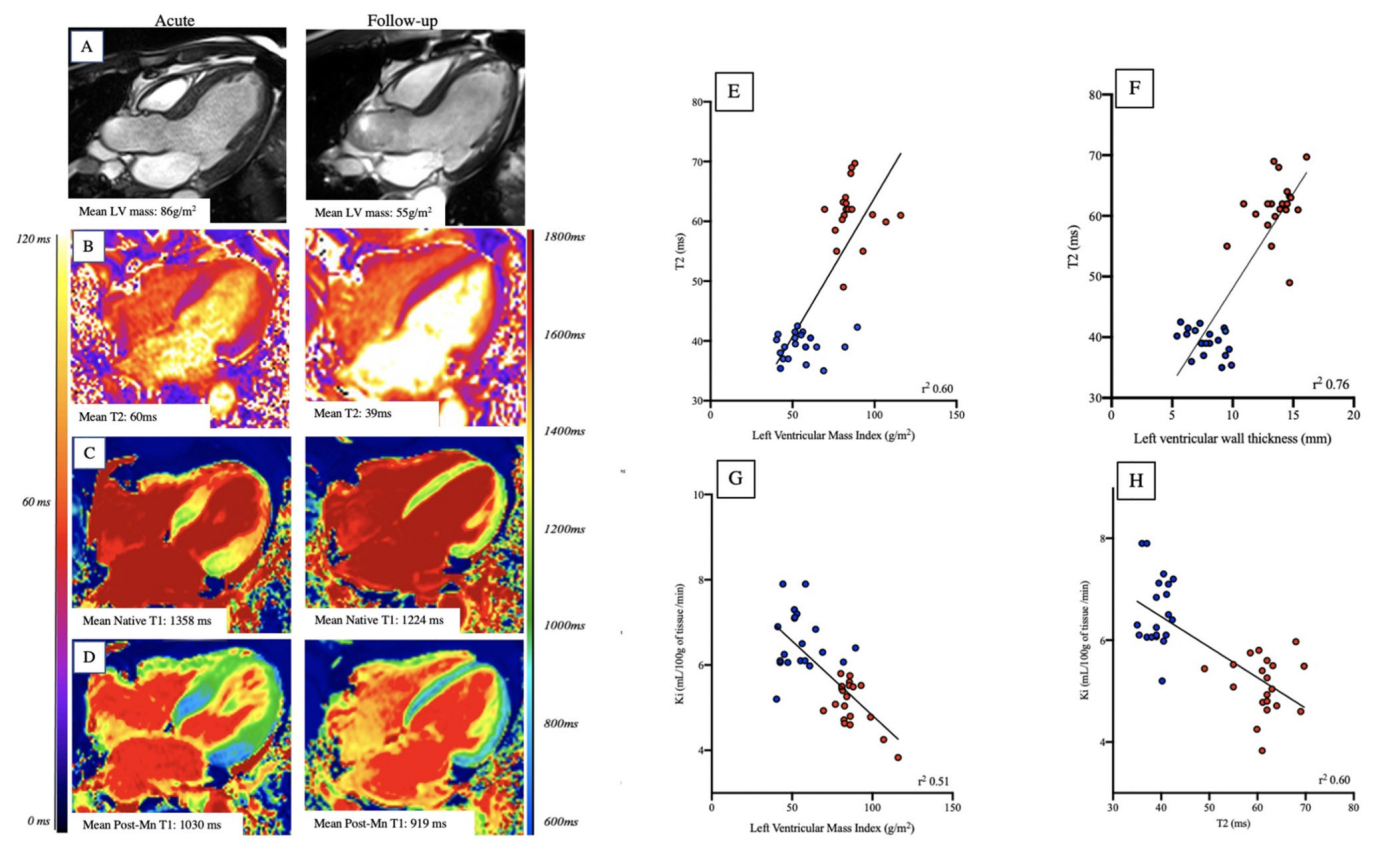
Myocardial Edema and Left Ventricular Mass in Takotsubo Syndrome Changes in left ventricular mass (panel A), native T2 (panel B), native T1 (panel C) and 30 min post manganese T1 maps (panel D). Correlations are seen between left ventricular mass and native T2 (E), left ventricular wall thickness and native T2 (F), myocardial manganese uptake and left ventricular mass (G) and myocardial manganese uptake and native T2 (H) during acute (red) and follow up (blue) scans.

**Figure 4:**
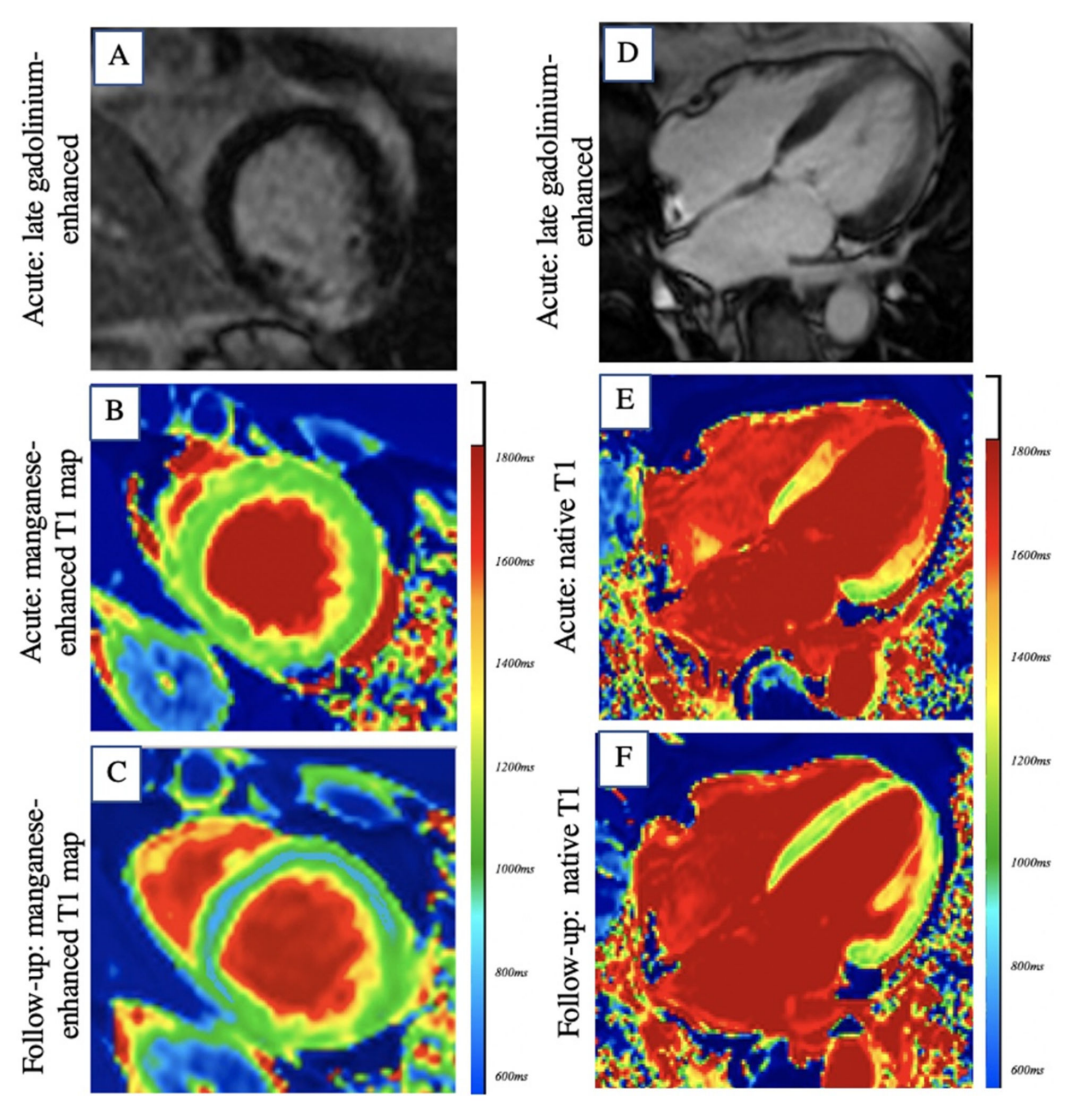
Gadolinium Enhancement in Patients with Takotsubo Syndrome Short-axis views of inferior late-gadolinium enhancement in a patient with spontaneous coronary artery dissection of the obtuse marginal branch of the left circumflex artery (A) and apical takotsubo (dual pathology). During acute imaging reduced myocardial manganese uptake (green) extends beyond the infarct region (B). Follow-up imaging demonstrates recovery of manganese uptake (blue) in regions affected by takotsubo syndrome with persistent abnormal manganese uptake (green) in the infarct region (C). Long-axis, four chamber view demonstrating characteristic hazy “incomplete nulling” in late-gadolinium enhancement imaging in a patient with apical takotsubo (D). Corresponding native T1 map during acute event demonstrates elevated native T1 in mid-ventricle and apical segments (E), with resolution on follow-up scans (F).

Most patients underwent cardiac magnetic resonance imaging within a median of 4 days (range 1 to 18 days) of symptom onset. Compared to matched control volunteers, patients with takotsubo syndrome had reduced left ventricular ejection fraction (Table 2), increased left ventricular mass (Figure 3) and comparable right ventricular systolic function. Interestingly left ventricular wall thickness was elevated in pathological and remote regions (Table 2). Two patients had late-gadolinium enhancement consistent with acute myocardial infarction (dual pathology, Figure 4). Two patients demonstrated hazy ‘incomplete nulling’ of late gadolinium enhancement imaging in mid-ventricular and apical segments consistent with the marked characteristic edema of takotsubo syndrome (Figure 4).

Compared to matched control volunteers, patients with takotsubo syndrome had elevated native T1 and T2 values in both the pathological and remote myocardial segments (Table 2). Following manganese infusion, T1 shortening was less pronounced in patients with takotsubo syndrome (Table 2) with kinetic modelling demonstrating marked reductions in myocardial manganese uptake in the pathological myocardium (5.1±0.5 versus 8.2±1.1 mL/100 g of tissue/min, P<0.0001). Segments with reduced myocardial manganese uptake corresponded with areas of myocardial edema and regional wall motion abnormalities. Subgroup analysis with the exclusion of the focal subtype of takotsubo syndrome (n=2; Table S5) or dual pathology (n=2; Table S6) demonstrated findings consistent with the entire cohort.

**Figure 5:**
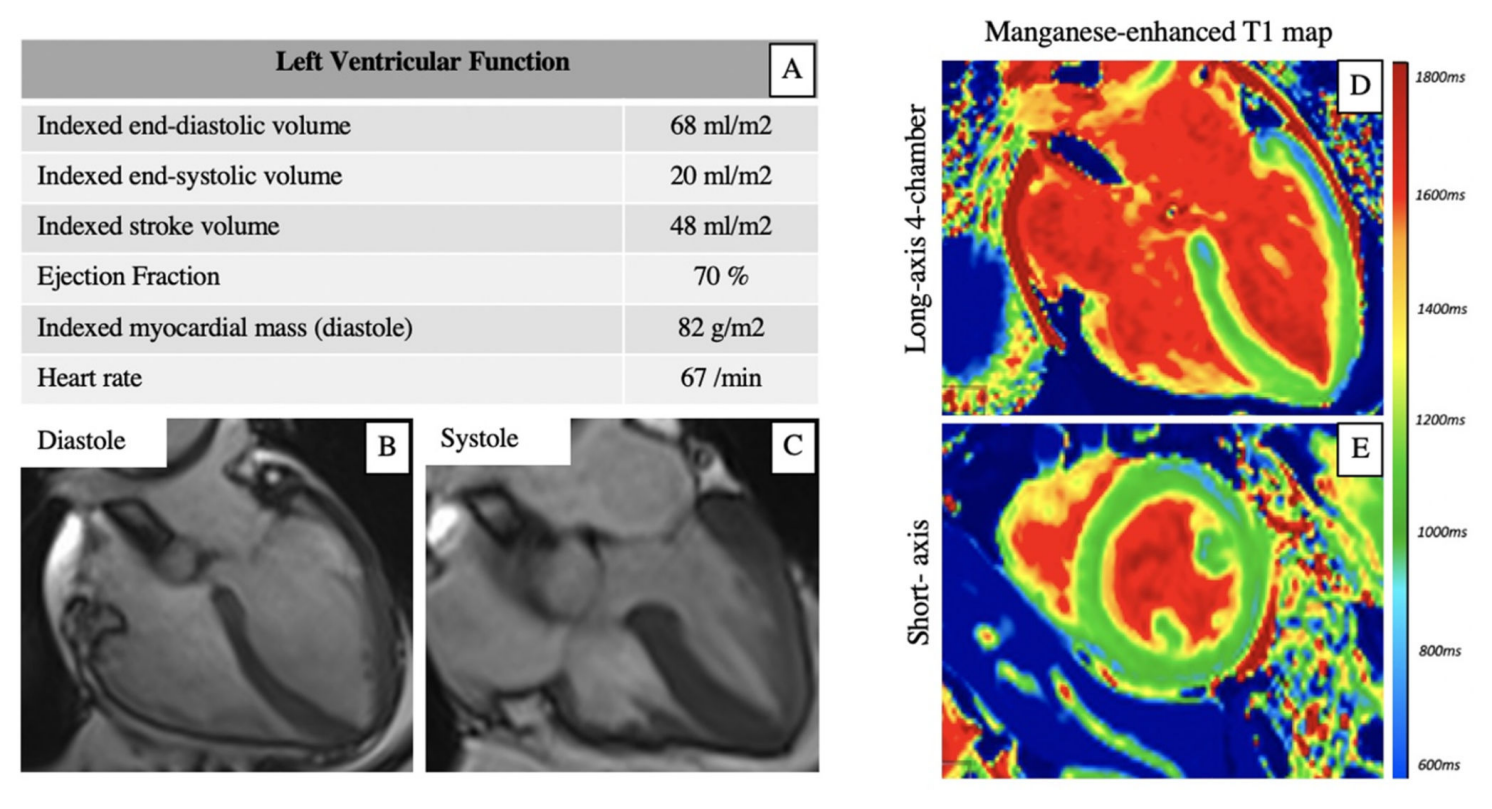
Manganese-enhanced Magnetic Resonance Imaging in Takotsubo Syndrome Resolution of left ventricular systolic dysfunction (A) and apical ballooning (B and C) in a patient with takotsubo syndrome scanned 18 days after symptom onset. Long-axis (D) and short-axis (E) manganese-enhanced T1 map demonstrating typical apical pattern of takotsubo syndrome with abnormal myocardial manganese uptake (green) in mid-ventricular and apical segments with normal uptake (blue) in basal segments despite apparent restoration of normal cardiac function.

One patient underwent cardiac magnetic resonance imaging 18 days after the onset of presentation symptoms, and there was complete resolution of regional wall motion abnormalities and normal left ventricular systolic function. Despite this, manganese-enhanced T1 mapping demonstrated a pattern of reduced myocardial manganese uptake consistent with apical takotsubo syndrome (Figure 5).

#### Follow Up at 3 Months

All 20 patients returned for follow-up and underwent repeat manganese-enhanced magnetic resonance imaging 100 days [median, range: 87-368] after symptom onset (Table 2) with 11 (65%) patients describing ongoing symptoms (Table 1). There was restoration of normal left ventricular ejection fraction and resolution of regional wall motion abnormalities in almost all patients (Table 2). Of the patients who were diagnosed with dual pathology (n=2), one demonstrated hypokinesis in the inferior wall despite normalization of left ventricular ejection fraction (acute myocardial infarction and takotsubo syndrome) and the other had resolution of their regional wall motion abnormality.

**Figure 6:**
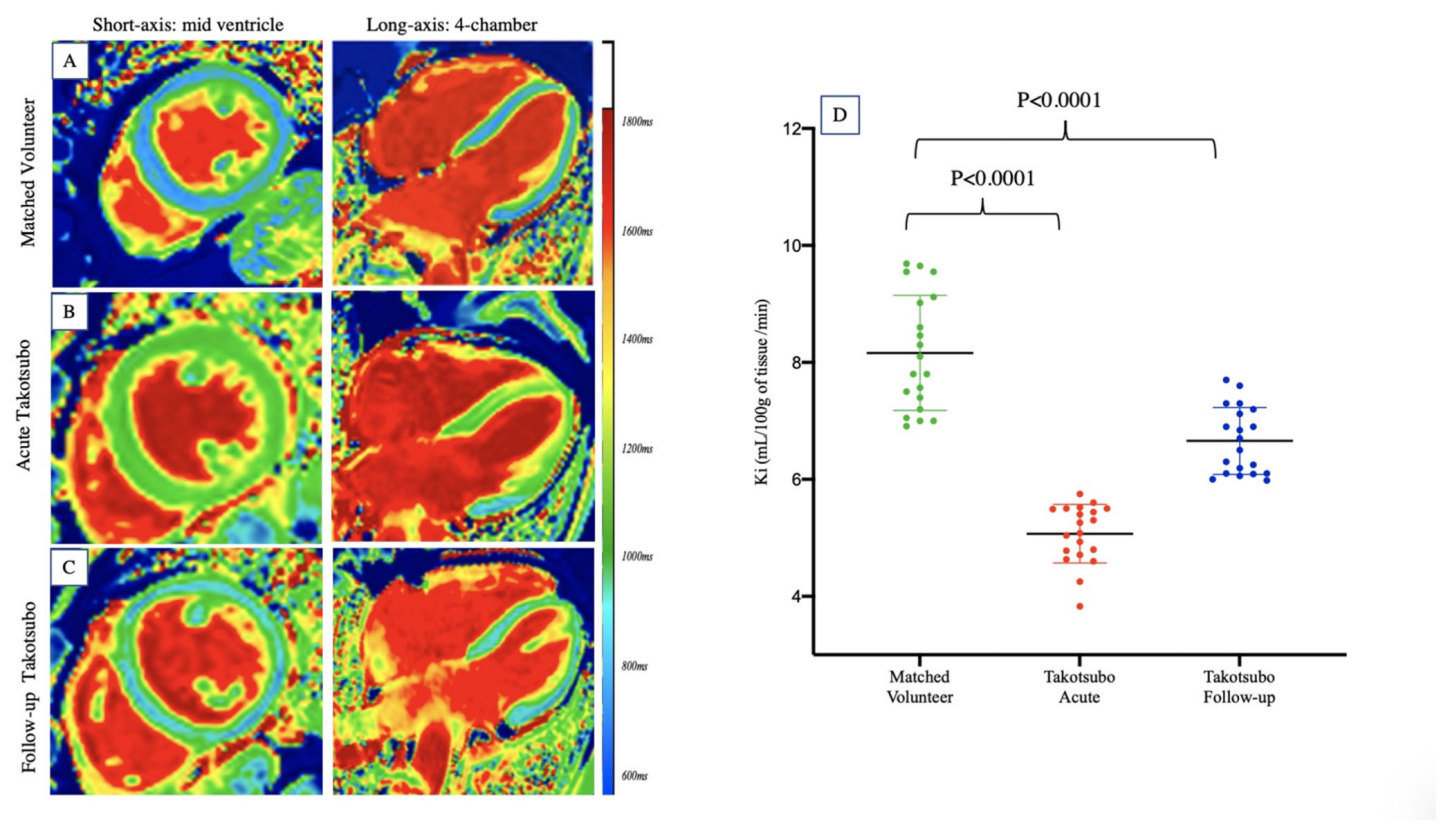
Myocardial Calcium Handling in Takotsubo Syndrome Short-axis and long-axis manganese-enhanced T1 map in a (panel A) matched control volunteer and (panel B) patient with acute takotsubo syndrome demonstrating reduced manganese uptake (green). Short-axis and long-axis manganese-enhanced T1 maps at follow-up (panel C) demonstrating improvement (patchy) myocardial manganese uptake (as calculated by Patlak modelling) but persistent abnormalities compared to matched control volunteers (D).

In patients with takotsubo syndrome, native and post-manganese T1 values were reduced compared to those obtained after the acute index event but remained higher than the matched control volunteers (P=0.02 and P=0.003 respectively). T2 values in the previously pathological myocardium normalized and were comparable to matched control volunteers (P=0.12). Left ventricular mass and left ventricular wall thickness (pathological and remote) were also reduced and correlated with the reduction in myocardial native T2 values (r^2^= 0.6 and 0.7 respectively, Figure 3). Myocardial manganese uptake demonstrated some improvement but remained persistently abnormal at follow-up (Table 2, Figure 6). Furthermore, manganese uptake correlated with improvement in left ventricular mass and T2 values (Figure 3). Subgroup analysis with the exclusion of the focal subtype of takotsubo syndrome (n=2; Table S5) or dual pathology (n=2; Table S6) demonstrated consistent findings.

## Discussion

We provide the first description of manganese-enhanced magnetic resonance imaging in detecting abnormal myocardial cellular physiology in patients with takotsubo syndrome. Our findings demonstrate that there is a profound disturbance in manganese uptake indicative of abnormal myocardial calcium handling. This is most marked in the acute setting but remains abnormal following apparent recovery of left ventricular function. This abnormal myocardial calcium handling may contribute to the underlying the pathophysiology of this condition, and perhaps explains persistent symptoms reported by some patients and the high rate of recurrence of takotsubo syndrome.^16–19^

For the first time, we have demonstrated a profound *in vivo* abnormality of myocardial calcium handling in patients with takotsubo syndrome. Our findings support previously described abnormalities of intracellular calcium handling in in endomyocardial biopsies obtained from patients with takotsubo syndrome and acute left ventricular dysfunction.^20^ Here, calcium-regulating proteins, such as phospholamban, sarcoendoplasmic reticulum calcium-adenosine triphosphatase (SERCA) and sarcolipin, were markedly altered suggesting that this may be responsible for the associated ventricular dysfunction. Using myocardial manganese uptake as a measure of the flux of intracellular calcium ions, we confirm that marked alterations in myocardial calcium handling appear to play a major pathophysiological role in takotsubo syndrome, especially in the acute high-risk period. Indeed, such a mechanism could explain why levosimendan can improve cardiogenic shock during acute severe cases of takotsubo syndrome since it augments myocardial calcium binding to troponin C. Although this is an important fundamental mechanistic observation, we cannot determine whether these alterations are a consequence or a cause of the takotsubo syndrome and this will require further study.

Takotsubo syndrome is partly defined by the apparent dramatic improvement of left ventricular function following the acute dramatic emergent presentation. The rapid transition from severe left ventricular systolic dysfunction and low ejection fraction to apparently normal left ventricular function and ejection fraction within days is a characteristic feature of this condition. However, this can create challenges for the diagnosis of this condition since unless the index of suspicion is high and the diagnosis is considered early, these typical features will resolve and the opportunity to undertake diagnostic imaging may have passed. In this regard, manganese-enhanced magnetic resonance imaging may provide an opportunity to identify the typical distribution of myocardial abnormalities despite normalization of regional wall motion abnormalities. This provides a unique opportunity to diagnose this condition at later time points and help resolve potential diagnostic uncertainties especially where the clinical suspicion of the diagnosis was initially low. There can also be major diagnostic uncertainty in cases of dual pathology where acute myocardial infarction triggers a takotsubo syndrome as demonstrated by two examples in our case series. Manganese-enhanced magnetic resonance imaging could therefore prove invaluable in identifying and discriminating cases of takotsubo syndrome, especially when the precipitating events is caused by another primary cardiac condition.

There are major implications for the protracted abnormalities in myocardial calcium handling. First, these findings could account for the persistence of symptoms and reduced exercise capacity reported by patients who have apparently recovered from takotsubo syndrome.^21^ The majority of patients continue to complain of fatigue, tiredness and reduced exercise tolerance despite the presence of a normal left ventricular ejection fraction. Continued long-term impairment in cardiac energetic status and reduced exercise maximal oxygen capacity has previously been described and are likely to be linked to the abnormalities of myocardial calcium handling.^21^ Indeed, 60% of our cohort described ongoing symptoms at follow up, compatible with a heart failure-like syndrome with a substantial impact on quality of life.

In contrast to the prior belief that the heart recovers spontaneously and completely without clinical sequelae, patients with takotsubo syndrome are now recognized to have substantial long-term morbidity and mortality which is comparable to that of acute myocardial infarction.^16, 19, 22–25^ Indeed, long-term rates of cerebrovascular and cardiac events and recurrent takotsubo syndrome approach 10% and 2% per year.^19, 24^ We have recently reported abnormal myocardial manganese uptake in patients with dilated or hypertrophic cardiomyopathy as well as those with acute myocardial infarction.^9^ Interestingly, levels of myocardial manganese uptake were more impaired in patients who had recovered from takotsubo syndrome than those with dilated cardiomyopathy despite the latter having more marked left ventricular systolic dysfunction.^9^ Indeed, such was the profound suppression of manganese enhancement during the acute phase of takotsubo syndrome that it was comparable to the infarcted myocardium of patients with acute myocardial infarction and the densely fibrotic regions in the left ventricle of patients with hypertrophic cardiomyopathy.

Whilst it would have been ideal to assess patients with takotsubo syndrome prior to their incident event, the longer-term persistence of abnormal myocardial calcium handling does suggest an underlying cardiomyopathy which is only brought to light following an acute stressful event. As such, we may never see normalization of myocardial calcium handling, and is again in keeping with previous studies describing a heart failure-like phenotype in this patient cohort.^21^

There are potential opportunities for manganese-enhanced magnetic resonance imaging to play an important role in prognostication and the assessment of novel treatment interventions. Persistent perturbation of myocardial calcium-handling may identify those patients who are at risk of incident or recurrent cases of takotsubo syndrome. Whilst this would seem intuitive, large prospective patient cohort studies are required to establish whether this is indeed the case. In addition, there are currently no proven treatments to improve the symptoms and clinical outcomes of patients with takotsubo syndrome. The assessment of myocardial calcium handling using manganese-enhanced magnetic resonance imaging could provide a very useful surrogate biomarker of treatment efficacy. This is particularly important since standard measures of cardiac function, such as left ventricular ejection fraction, appears to be normal in patients who have recovered from takotsubo syndrome, and this has limited the field in terms of assessing the efficacy of potential preventative therapeutic interventions. It is noteworthy that persistent abnormalities of myocardial calcium handling were present despite the initiation of angiotensin converting enzyme inhibitor or beta-blocker therapies in many of our patients.

Our study has several limitations that should be acknowledged. First, there have been prior concerns regarding toxicity of unchelated forms of manganese. However, the chelated form of manganese used here retains the necessary properties for intracellular myocardial manganese uptake without any demonstrable adverse hemodynamic or arrhythmic effects.^26, 27^ Second, our study population size was modest, although we detected substantial and large abnormalities in myocardial calcium handling. Future multicenter studies of larger patient populations are needed to demonstrate the robustness, generalizability and clinical utility of our findings in this proof-of-concept study. Third, the Patlak model assumes that perfusion is normal. Whilst we excluded epicardial coronary flow disturbance on invasive angiography in all patients, microvascular impairment and reduced myocardial perfusion may have contributed to reductions in manganese uptake. However, although microvascular impairment is a potential feature in takotsubo syndrome,^28^ this abnormality improves as myocardial function normalizes^29^ and we observed persistent long-term abnormalities of myocardial manganese uptake following apparent recovery. This suggests that microvascular impairment alone is unlikely to be the cause of the marked myocardial manganese abnormality seen in our takotsubo syndrome cohort. Finally, there are no currently available preparations of manganese-based contrast medium for widespread clinical use. However, this is likely to change with commercially available preparations anticipated in the near future.

In conclusion, we have conducted the first proof-of-concept study of manganese-enhanced magnetic resonance imaging in patients with takotsubo syndrome. Using kinetic modelling, we have observed dysfunctional myocardial calcium influx in patients with takotsubo syndrome which is most striking during the acute episode but persists despite improvement of edema and myocardial function. We believe that manganese-enhanced magnetic resonance imaging holds major promise for the diagnosis, risk stratification and monitoring of disease, with the potential for the assessment of novel treatment interventions.

## Supplementary Material

Supplemental Publication Material

## Figures and Tables

**Table 1:** Baseline characteristics of the study populations.

	Matched Control Subjects (n=20)	Patients with Takotsubo syndrome (n=20)	P value
**Age** (years)	59 [29-70]	63 [42-80]	0.1
**Female sex**	14 (70)	18 (90)	
**Body Mass Index** (kg/m^2^)	27 [21-35]	25 [22-33]	0.67
**Past Medical History**			
Hypertension	7(35)	8 (40)	
Known coronary artery disease	2(10)	2 (10)	
Hypercholesterolemia	5 (25)	5 (25)	
Acute neurological/psychiatric disorder	0	1 (5)	
Past of chronic neurological/psychiatric disorder	4 (20)	14 (70)	
Depression Anxiety disorder Schizophrenia	3 (15) 1 (5) 0	7 (35) 6 (30) 1 (5)	
Diabetes Mellitus	2 (10)	2 (10)	
Thyroid disorder	0	2 (10)	
Asthma	0	1 (5)	
**Medications prior to admission**			
Antiplatelet therapy	2 (10)	2 (10)	
Beta-Blocker therapy	2 (10)	2 (10)	
ACE inhibitor or ARB therapy	4 (35)	2 (10)	
Calcium channel blockers	1 (10)	5 (25)	
Statin therapy	5 (25)	5 (25)	
Anti-glycemic therapy	2 (10)	2(10)	
Previous/Current Antidepressant therapy	2 (10)	9 (45)	

**Presenting symptom**	-		
Dyspnea	-	6 (30)	
Chest pain	-	14 (70)	
**Stressor**	-		
Emotional	-	13 (65)	
Physical	-	4 (20)	
None	-	3 (15)	
**Peak plasma cardiac high sensitive troponin I concentration (ng/L)**	-	6,981	
**ECG**	-		
ST-segment elevation	-	8 (40)	
ST-segment depression	-	2 (10)	
T-wave changes	-	10 (50)	
QTc (ms)	387 ± 13	552 ± 42	
**Index left ventricular ejection fraction**	-		
Borderline (50-54%)	-	4 (20)	
Impaired (36-49%)	-	9 (45)	
Severely impaired <35%)	-	7 (35)	
**Coronary Angiography**	-	20 (100)	
Normal coronaries	-	10 (50)	
Non-obstructive arteries	-	10 (50)	
Obstructive disease	-	0	
**Left ventriculography completed**	-	13 (65)	
**Takotsubo syndrome sub-type**	-		
Apical	-	17 (85)	
Basal	-	1 (5)	
Focal	-	2(10)	
**Treatment initiated/stopped**	-		
Antiplatelet therapy	-	10 (50)	
Beta-Blocker therapy	-	18 (90)	
ACE inhibitor or ARB therapy	-	16 (80)	
Diuretic therapy	-	6 (30)	
Statin therapy	-	5 (25)	
Calcium channel antagonist^*^	1 (10)	5 (25)	

**Days since acute episode**	-	100 [87-368]	
**Symptoms at follow-up**	-	11 (61)	
Chest pain	-	0 (0)	
Dyspnea	-	5 (28)	
Palpitations	-	2 (11)	
Fatigue	-	4 (20)	
**Adverse events at follow-up**	-		
Cerebrovascular event	-	1 (5)	
Reoccurrence	-	1 (5)	
Death	-	0 (0)	

Number (%), median [interquartile range], mean ± standard deviation

* Withdrawn at presentation in patients or for at least 48h prior to manganese-enhanced magnetic resonance imaging.

**Table 2:** Cardiac magnetic resonance findings

	Matched Control (n=20)	Patients with takotsubo syndrome: index event (n=20)	Patients with takotsubo syndrome: follow-up (n=20)	P value^*^	P value^**^
Time between symptom onset and scan (median, days)	-	4 [1-18]	100 [87-368]		
Left ventricular end-diastolic volume index (mL/m^2^)	79±15	71±20	72±11	0.1	0.09
Left ventricular end-systolic volume index (mL/m^2^)	27±7	36±11	23±9	**0.03**	0.89
Stroke volume index (mL/m^2^)	51±11	39±12	46±7	**0.002**	0.83
Left ventricular ejection fraction (%)	67±8	51±11	69±4	**<0.001**	0.61
Left ventricular mass index (g/m^2^)	57±14	86±11	55±13	**<0.001**	0.71
Left ventricular wall thickness- pathological (mm)	7.0±0.9	13.1±1.5	7.3±1.3	**<0.001**	0.12
Left ventricular wall thickness- remote (mm)	6.9±0.9	10.4±1.8	7.0±1.1	**<0.001**	0.09
Right ventricular ejection fraction (%)	63±6	63±10	66±4	0.47	0.09
Global longitudinal strain (%)	-18±1	-12±6	-16±3	**0.003**	0.09
Late-gadolinium enhancement pattern	0 (0)	2 (10)	-		
Ischemic	-	2 (10)	-		
Native T1 in pathological segment (ms)	1211±28	1358±49	1238±35	**<0.0001**	**0.02**
Native T1 in remote segment ^(ms)^	1211±28	1255±56	1209±27	**0.01**	0.84
Native T2 in pathological segment (ms)	38±3	60±7	39±2	**<0.0001**	0.12
Native T2 in remote segment ^(ms)^	38±3	43±5	36±3	**0.002**	0.24
Global extracellular volume (%)	26±3	34±5	-	**<0.0001**	-
Myocardial T1 30 min after manganese pathological segment (ms)	884±26	1030±48	919±31	**<0.0001**	**0.003**
Myocardial T1 30 min after manganese remote segment ^(ms)^	884±26	920±16	900±9	**<0.0001**	**0.002**
Manganese influx (Ki; mL/100 g of tissue min)	8.2±1.1	5.1±0.5	6.6±0.5	**<0.0001**	**<0.0001**

Number (%), median [interquartile range], mean ± standard deviation

* Controls v patients with takotsubo syndrome at index presentation.

** Controls v patients with takotsubo syndrome at follow-up

## Clinical Perspectives

### What is new?

- In patients with takotsubo syndrome, characteristic patterns of myocardial manganese enhancement can be demonstrated on magnetic resonance imaging.
- Patients with takotsubo syndrome have both acute and persistently abnormal myocardial manganese uptake consistent with underlying abnormalities of myocardial calcium handling.
- Despite apparent restoration of normal systolic function, abnormalities of myocardial calcium handling persist for at least 3 months.

### What are the clinical implications?

- Manganese-enhanced magnetic resonance imaging has the potential to diagnose and to characterize takotsubo syndrome even after apparent restoration of normal cardiac function.
- Abnormal myocardial calcium handling as demonstrated by reduced manganese uptake may underlie the pathophysiology of takotsubo syndrome and manganese-enhanced magnetic resonance imaging potentially provides a measure of disease severity.
- Persistent abnormalities of myocardial calcium handling may explain the associated long-term morbidity and mortality of takotsubo syndrome.

## Non Standard Abbreviations and Acronyms

MRI: magnetic resonance imaging
EF: ejection fraction
