# Supplementary material for: Manganese-Enhanced Magnetic Resonance Imaging in Takotsubo Syndrome
Source: Circulation. Author manuscript; Available in PMC 2022 Dec 13. (PMC7613919; doi:10.1161/CIRCULATIONAHA.122.060375)
Supplement: Supplemental Publication Material [file EMS155924-supplement-Supplemental_Publication_Material.pdf]

**Supplementary Material**

# Manganese-Enhanced Magnetic Resonance Imaging in Takotsubo Syndrome

Singh T BM, [1,2,3] Joshi S MBBS, [1,2,3] Kershaw L PhD, [1,3]

Baker AH BSc PhD,[1,2,3] McCann GP MBChB MD,[5] Dawson DK MD DPhil,[4]

Dweck MR MBChB PhD,[1,2,3] Semple SI MSc PhD,[1,3*] Newby DE DM PhD [1,2,3*]

1. BHF/University Centre for Cardiovascular Science, University of Edinburgh, UK

2. Edinburgh Heart Centre, Royal Infirmary of Edinburgh, UK

3. Edinburgh Imaging, University of Edinburgh, UK

4. Aberdeen Cardiovascular and Diabetes Centre, University of Aberdeen, Aberdeen, UK

5. Department of Cardiovascular Sciences, University of Leicester and NIHR Leicester Biomedical Research Centre, Glenfield Hospital, UK

**Supplementary Figure 1**
**Manganese-enhanced magnetic resonance imaging protocol**

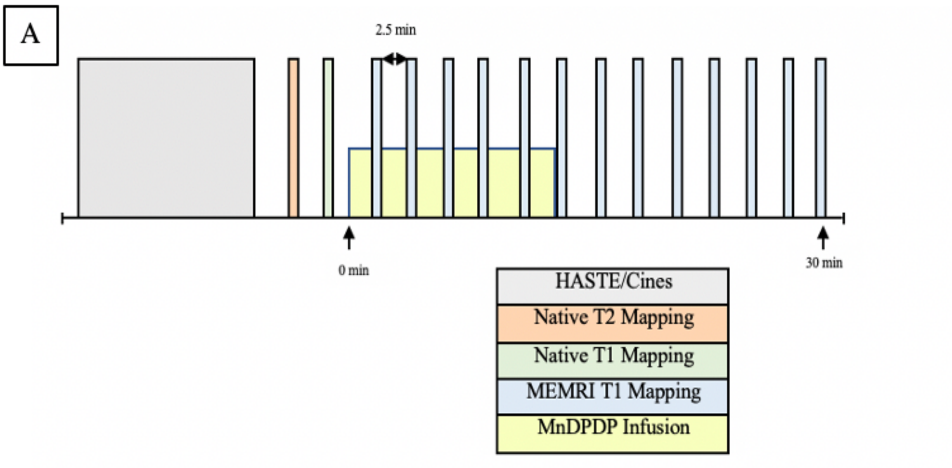


MEMRI, Manganese-enhanced magnetic resonance imaging, MnDPDP, manganese dipyridoxyl diphosphate

**Supplementary Figure 2**

**T1 Mapping of manganese-enhanced magnetic resonance imaging**

T1 maps 30 min after manganese-based contrast media administration demonstrating regions of interest in pathological myocardium (blue semicircle) in a matched volunteer (A) and a patient with apical (B), basal (C) and focal (D) takotsubo syndrome.

BP, bloodpool

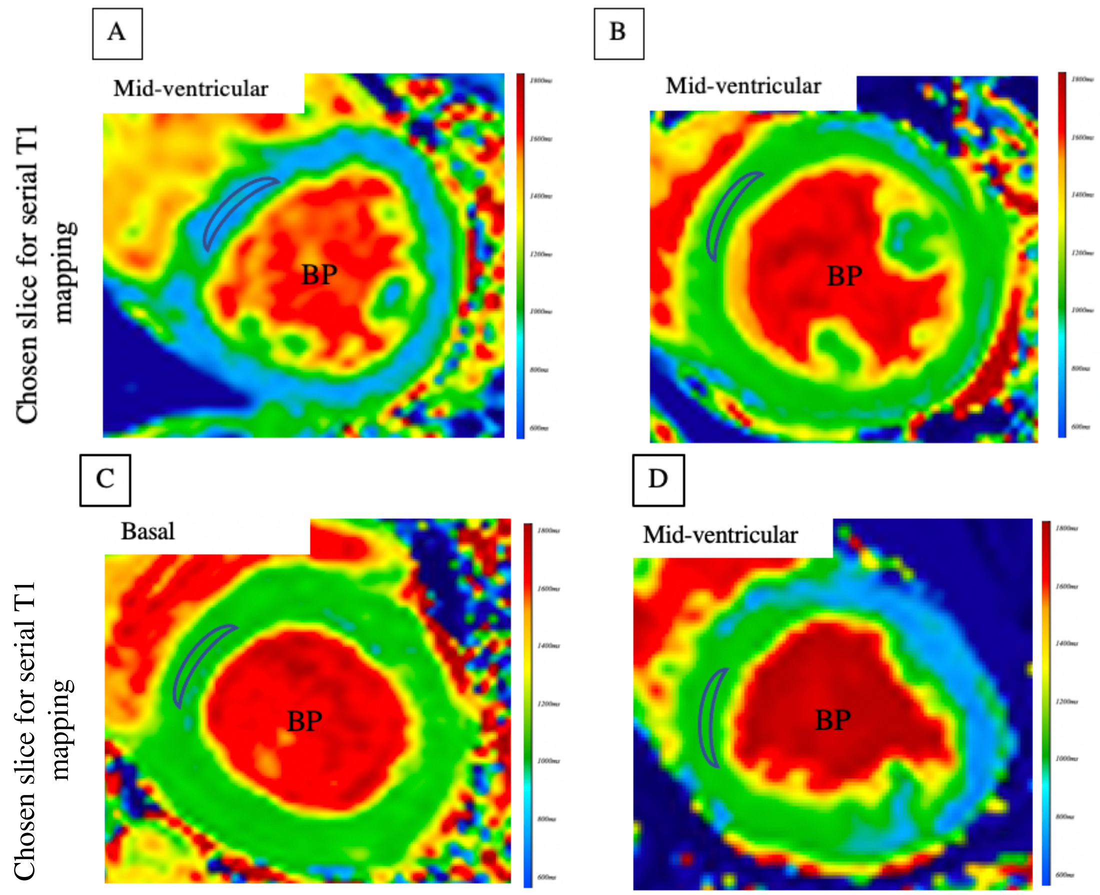

**Supplementary Figure 3**
**Left ventricular edema in patients with takotsubo syndrome**
Left ventricular wall thickness measurement in remote and pathological myocardium in a patient with takotsubo syndrome during acute (panel A) and follow-up (panel B).

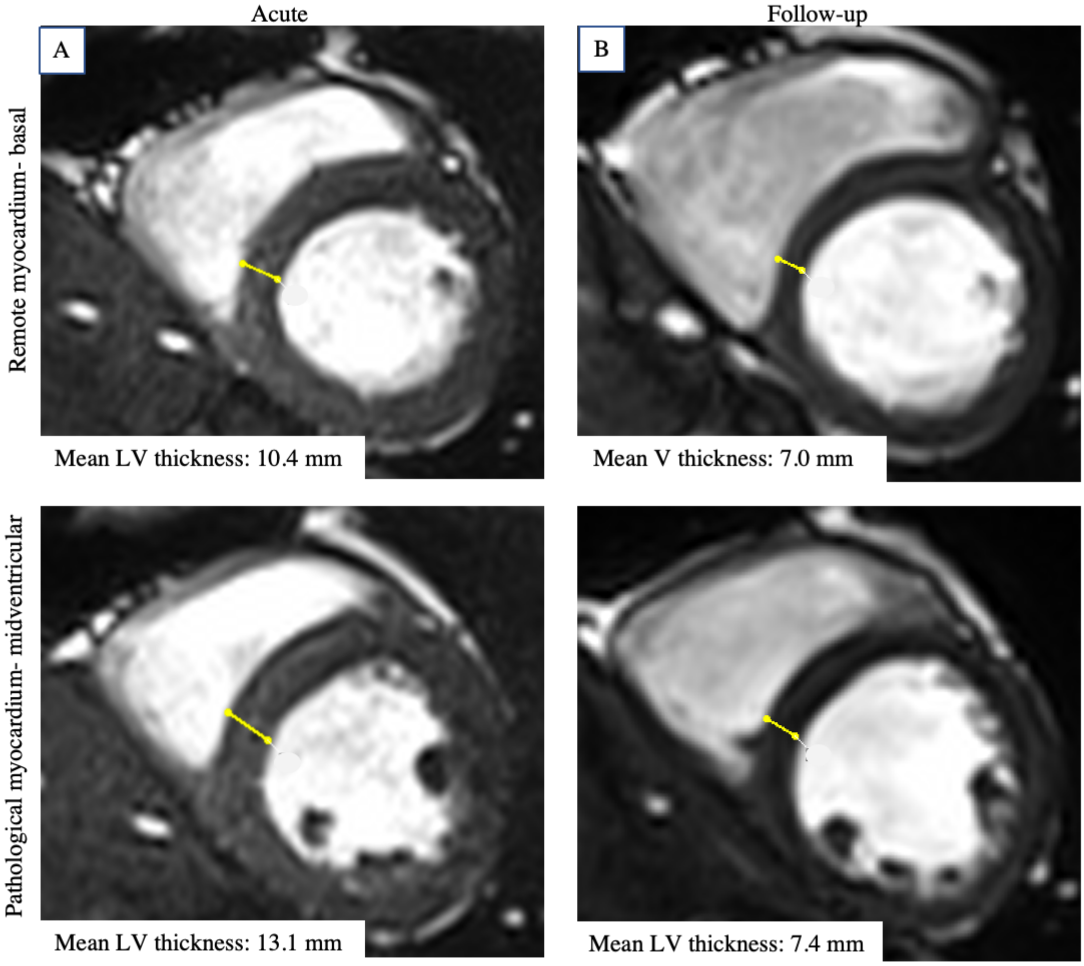


LV, left ventricle

**Supplementary Figure 4 Haemodynamic and Electrocardiography monitoring with manganese-enhanced magnetic resonance imaging.**

Blood pressure and heart rate after administration of manganese dipyridoxyl diphosphate in matched control, (A) and patients with takotsubo syndrome (B).

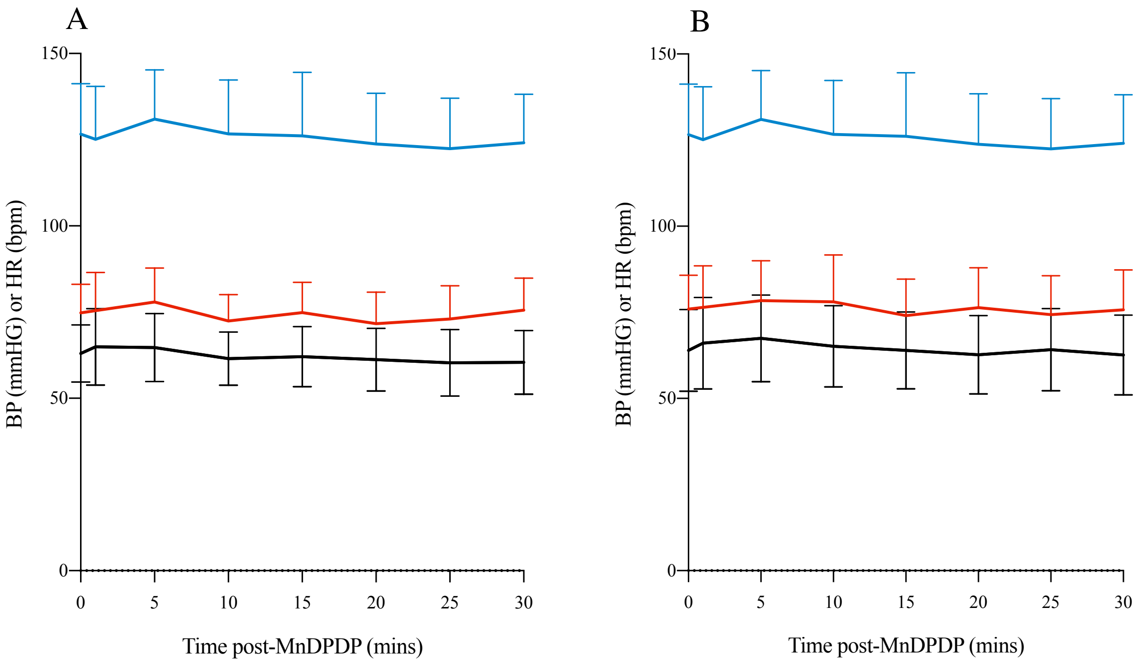


MnDPDP, manganese dipyridoxyl diphosphate, SBP, systolic blood pressure, DBP, diastolic blood pressure, HR, heart rate.

**Supplementary Table 1: Global native T1, post-manganese T1 and myocardial manganese uptake (Ki) values**

| | **Matched Control (n=20)** | **Patients with takotsubo syndrome: index event (n=16)** † | **Patients with takotsubo syndrome: follow-up (n=16)** † | **P value*** | **P value**** |
|---|---|---|---|---|---|
| Native T1 (ms)- Septal | 1211±28 | 1358±49 | 1237±30 | **<0.0001** | **0.03** |
| Native T1 (ms)- Global | 1212±28 | 1360±59 | 1231±23 | **<0.0001** | **0.04** |
| Myocardial T1 30 min after manganese (ms)- Septal | 884±26 | 1030±48 | 914±22 | **<0.0001** | 0.02 |
| Myocardial T1 30 min after manganese (ms)- Global | 892±22 | 1222±31 | 908±21 | **<0.0001** | **0.01** |
| Manganese influx (Ki; mL/100 g/tissue min)- Septal | 8.0±1.0 | 5.1±0.5 | 6.8±0.6 | **<0.0001** | **<0.001** |
| Manganese influx- (Ki; mL/100 g/tissue min)- Global | 8.2±1.1 | 5.1±0.6 | 6.7±0.5 | **<0.001** | **0.001** |

*Controls v patients with takotsubo syndrome at index presentation
** Controls v patients with takotsubo syndrome at follow-up.
† Patients with focal takotsubo and dual pathology excluded (n=16)

**Supplementary Table 2: Precipitating stressors in patients with takotsubo syndrome**

| **Type of stressor** | **Example** |
|---|---|
| **Emotional (n=13)** | |
| | Bereavement of family member/friend (x4) |
| | Bereavement of family pet (came home to find dog had attacked cat) |
| | Argument with husband |
| | Argument with daughter-in-law |
| | Argument with friend (x2) |
| | Receiving hostile email from landlord |
| | Driving the wrong way on the motorway |
| | Workplace stress (x2) |
| **Physical (n=4)** | |
| | Endoscopy procedure |
| | Acute cholecystitis |
| | Loch swimming |
| | Direct current cardioversion procedure |

**Supplementary Table 3: Myocardial manganese uptake (Ki) during acute and follow up takotsubo syndrome by pre-existing psychiatric, neurological disorder or anti-depressant use.**

| Myocardial Manganese uptake (Ki) mL/100 g of tissue/min | Patients with pre-existing psychiatric, neurological disorders or antidepressant use (n=9) | Patients without pre-existing psychiatric, neurological disorders or antidepressant use (n=11) | P value |
|---|---|---|---|
| Acute event | 5.0± 1.1 | 5.1±0.9 | 0.72 |
| Follow-up | 6.7±0.8 | 6.6±1.0 | 0.53 |

**Supplementary Table 4**
**Coronary angiography and left ventriculography in patients with takotsubo syndrome**

| ID | Sex | Age | Diagnosis | Coronary Angiography | | Left ventriculography | |
|---|---|---|---|---|---|---|---|
| | | | | Right coronary system | Left coronary system | Diastole | Systole |
| 1 | M | 39 | Basal takotsubo | 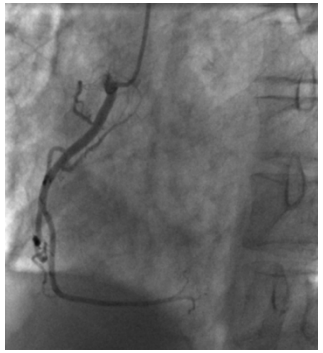 | 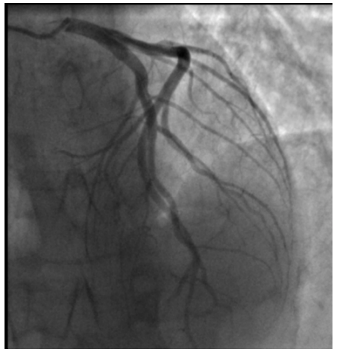 | 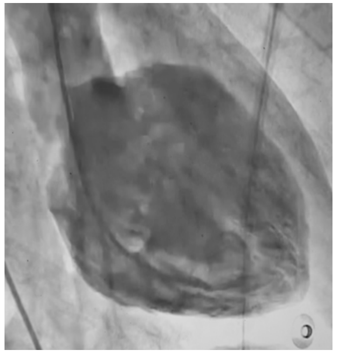 | 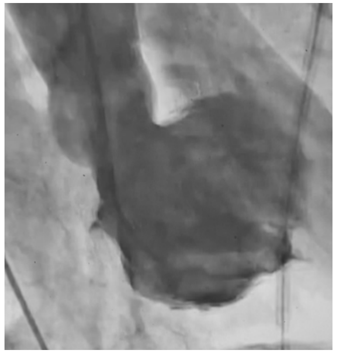 |
| 2 | F | 68 | Focal takotsubo | 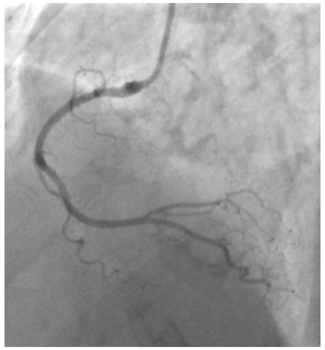 | 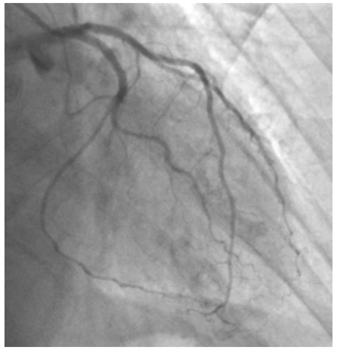 | Not performed | |

| | | | | | | | |
|---|---|---|---|---|---|---|---|
| 3 | F | 57 | Focal takotsubo | 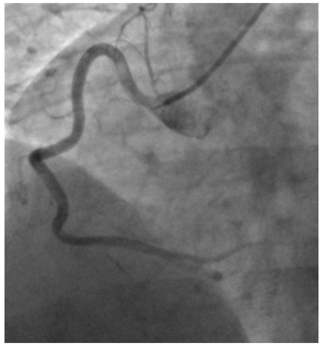 | 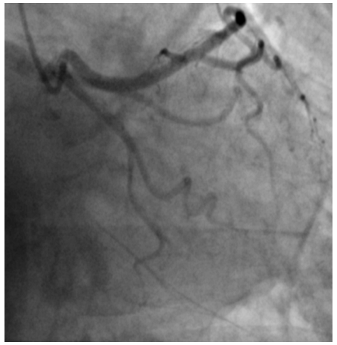 | 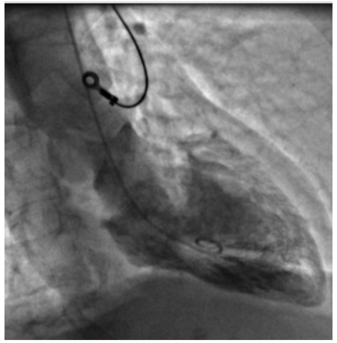 | 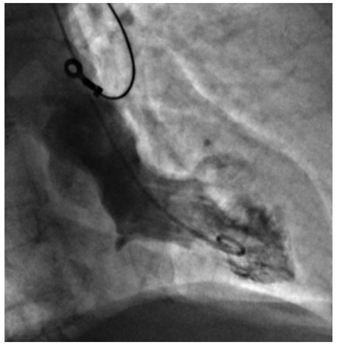 |
| 4 | F | 65 | Apical takotsubo | 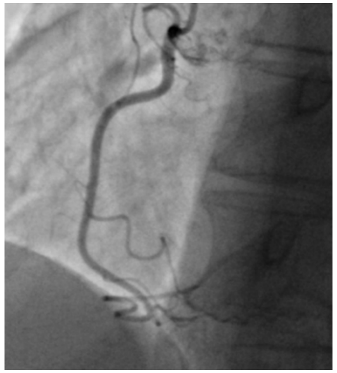 | 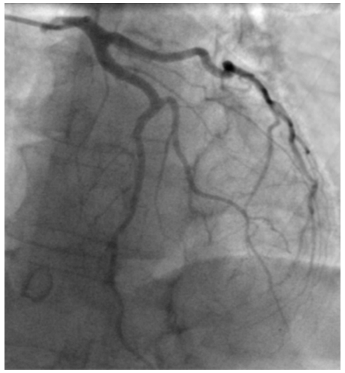 | 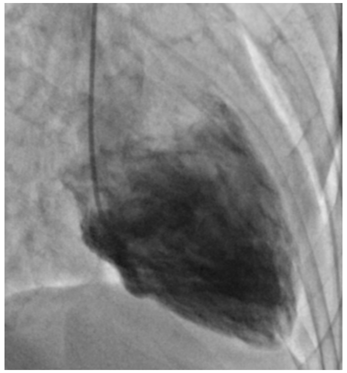 | 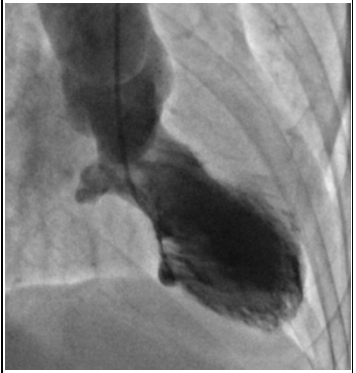 |

| | | | | | | | |
|---|---|---|---|---|---|---|---|
| 5 | F | 40 | Apical takotsubo | 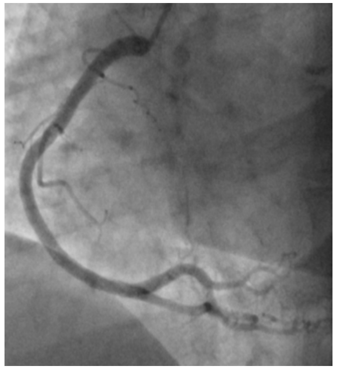 | 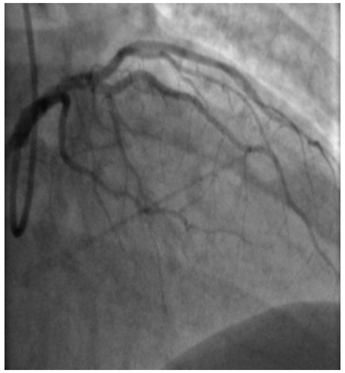 | 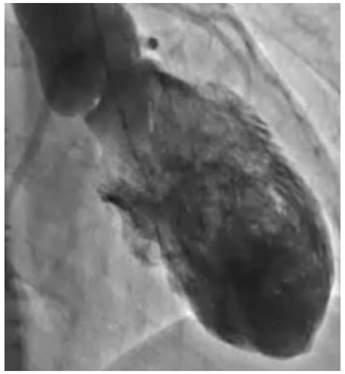 | 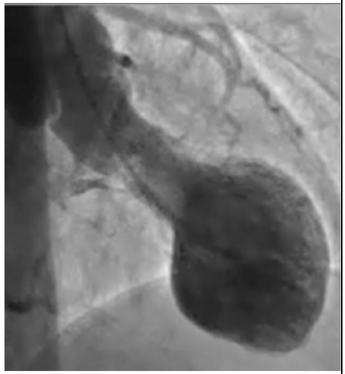 |
| 6 | F | 50 | OM SCAD and apical takotsubo | 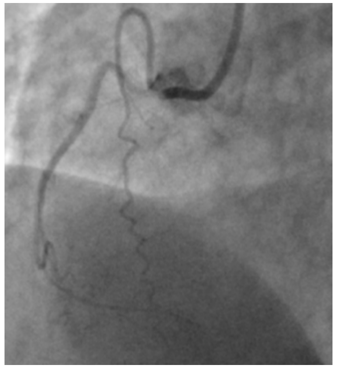 | 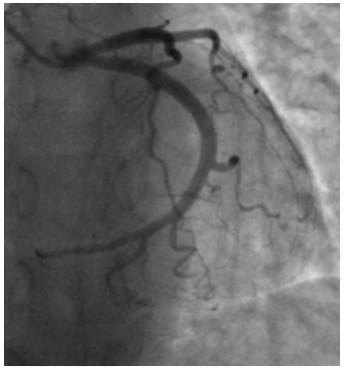 | Not performed | |

| | | | | | | | |
|---|---|---|---|---|---|---|---|
| 7 | F | 59 | Apical takotsubo | 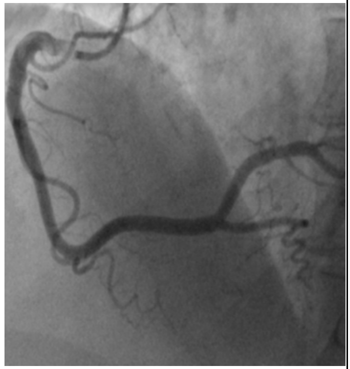 | 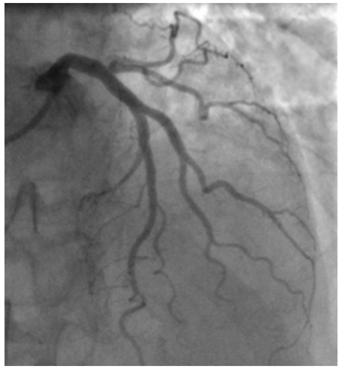 | Poor contrast injection and opacification | |
| 8 | F | 60 | Apical takotsubo | 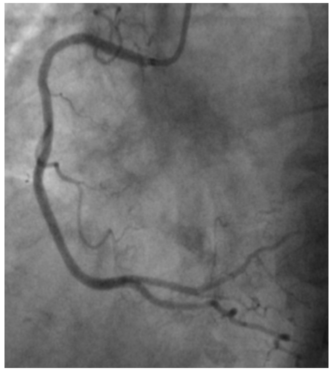 | 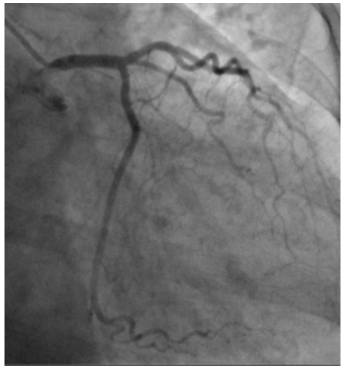 | 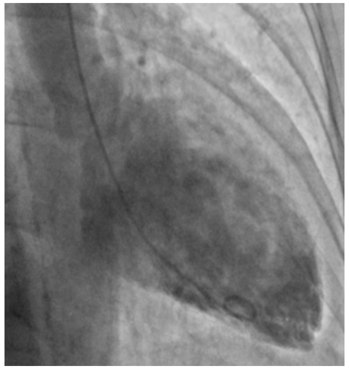 | 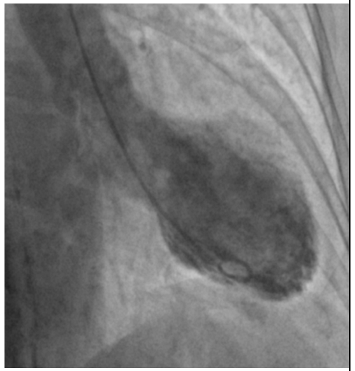 |

| | | | | | | | |
|---|---|---|---|---|---|---|---|
| 9 | F | 61 | Apical takotsubo | 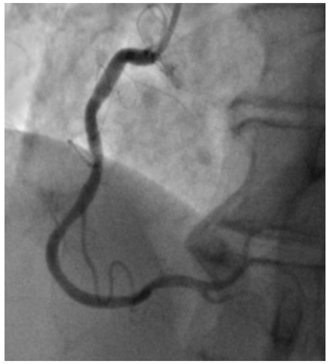 | 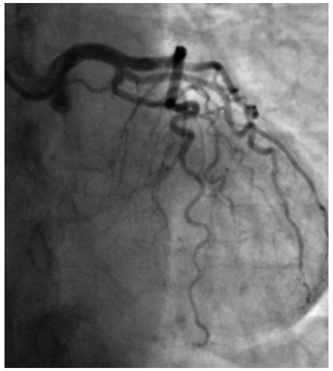 | 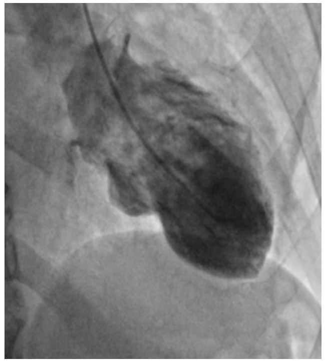 | 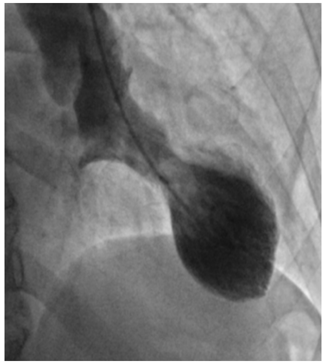 |
| 10 | M | 49 | Apical takotsubo | 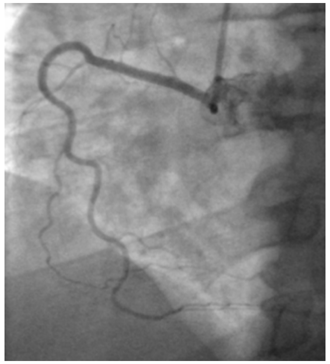 | 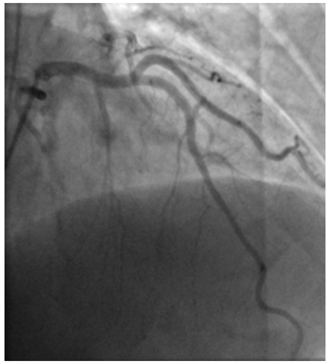 | Not performed | |

| | | | | | | | |
|---|---|---|---|---|---|---|---|
| 11 | F | 56 | Apical takotsubo | 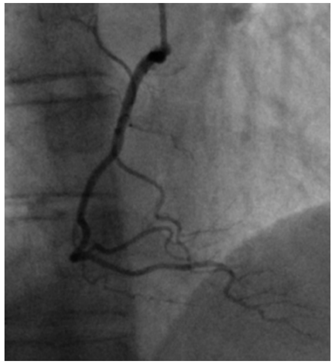 | 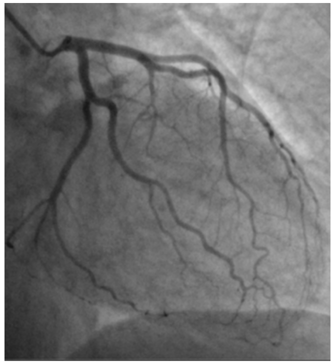 | Not performed | |
| 12 | F | 59 | Apical takotsubo | 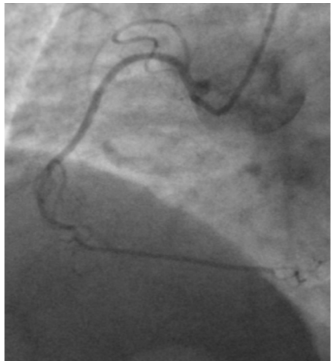 | 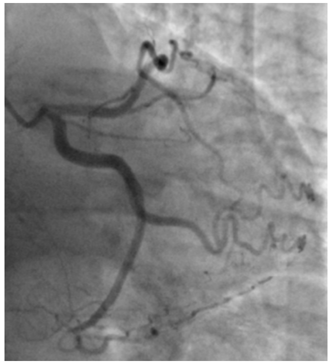 | 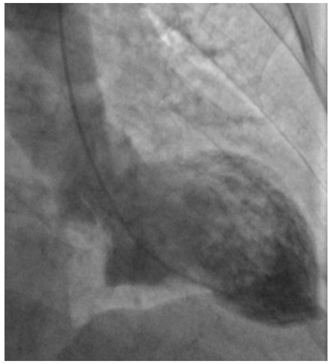 | 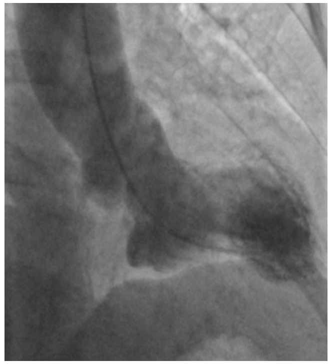 |

| | | | | | | | |
|---|---|---|---|---|---|---|---|
| 13 | F | 66 | Apical takotsubo | 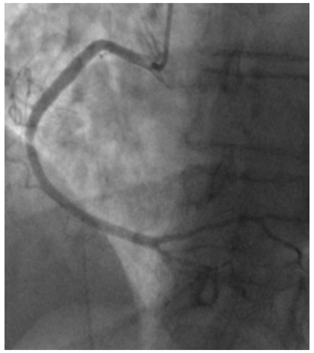 | 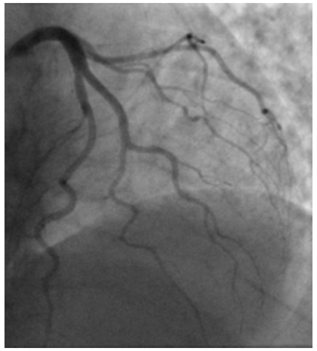 | 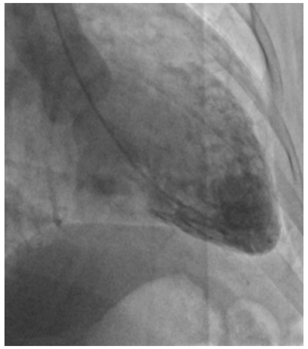 | 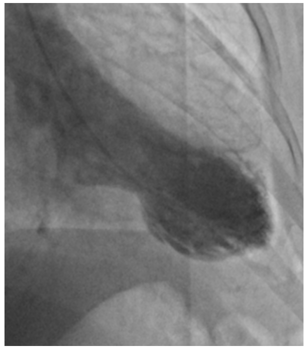 |
| 14 | F | 66 | Apical takotsubo | 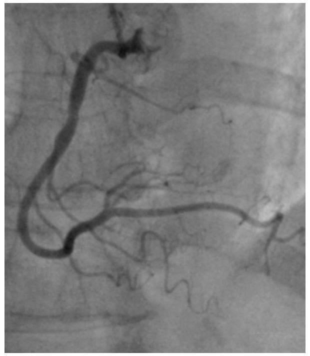 | 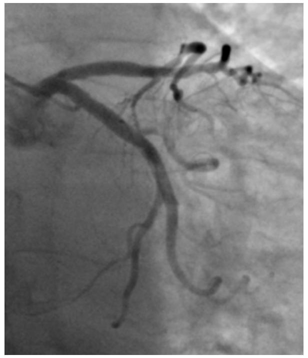 | 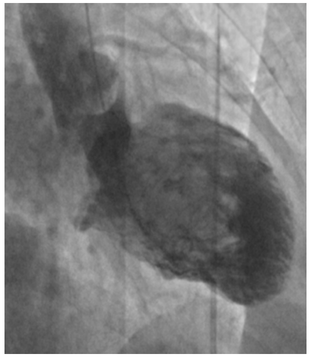 | 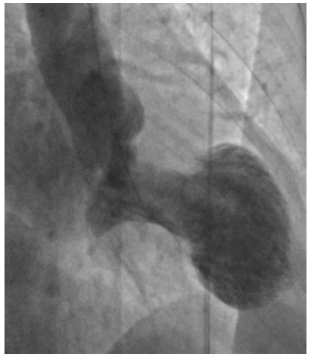 |

| | | | | | | | |
|---|---|---|---|---|---|---|---|
| 15 | F | 50 | Apical takotsubo | 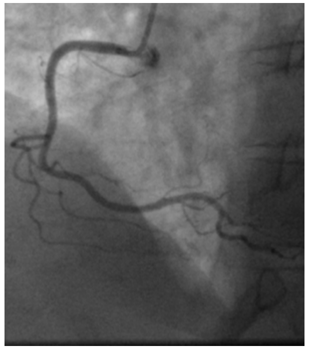 | 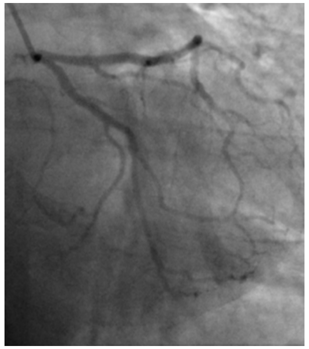 | Not performed | |
| 16 | F | 62 | Apical takotsubo | 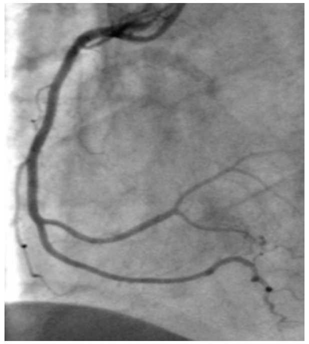 | 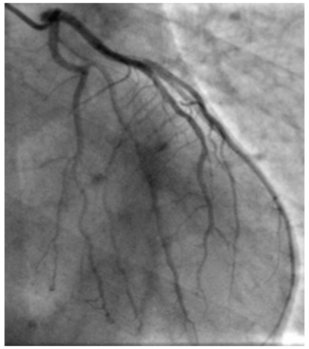 | Not performed | |

| | | | | | | | |
|---|---|---|---|---|---|---|---|
| 17 | F | 38 | Apical takotsubo | 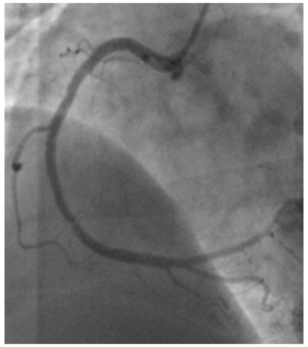 | 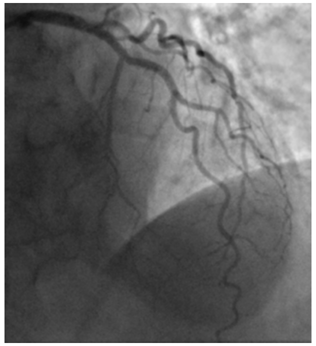 | 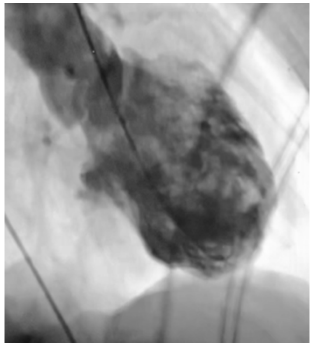 | 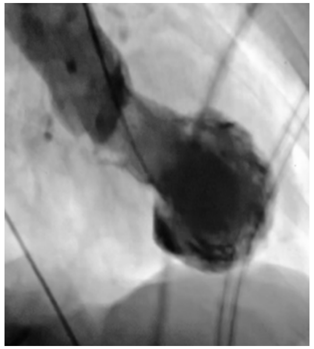 |
| 18 | F | 67 | Apical takotsubo | 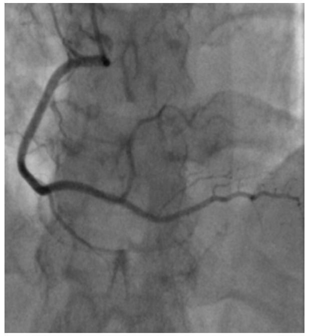 | 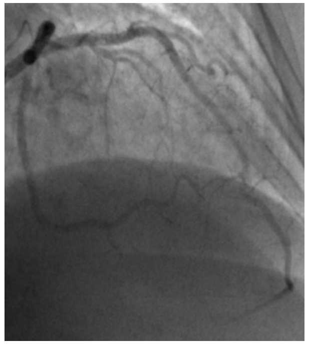 | 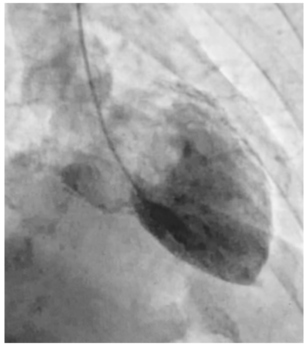 | 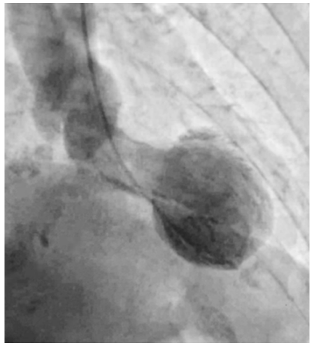 |

| | | | | | | | |
|---|---|---|---|---|---|---|---|
| 19 | F | 60 | Apical takotsubo | 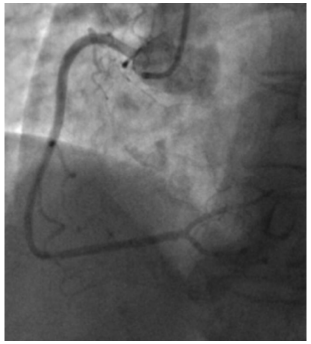 | 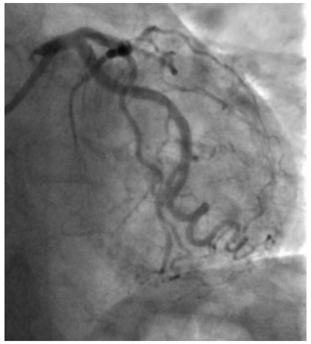 | Not performed | |
| 20 | F | 67 | Apical takotsubo | 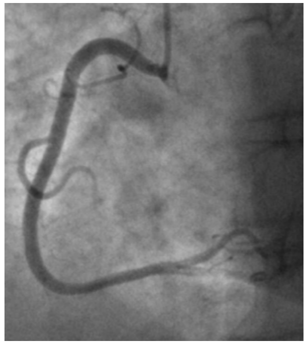 | 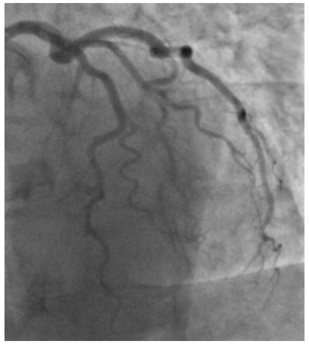 | 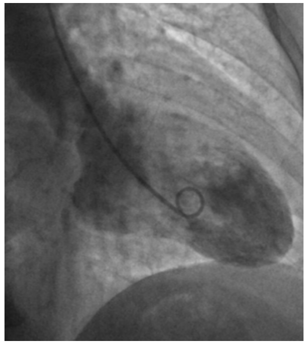 | 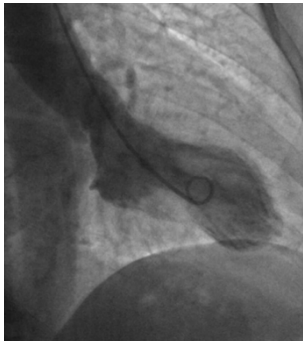 |

OM, Obtuse marginal, SCAD, Spontaneous coronary artery dissection

**Supplementary Table 5: Native T1, post-manganese T1 and myocardial manganese uptake (Ki) in all patients and in takotsubo syndrome excluding focal takotsubo.**

| | **Matched Control (n=20)** | **All takotsubo syndrome (n=20)** | | **P value*** | **P value**** | **Focal Takotsubo excluded (n=18)** | | **P value†** | **P value††** |
|---|---|---|---|---|---|---|---|---|---|
| | | Acute | Follow up | | | Acute | Follow up | | |
| Native T1 (ms)- Septal | 1211±28 | 1358±49 | 1238±35 | **<0.0001** | **0.02** | 1361±75 | 1237±36 | **<0.0001** | **0.04** |
| Myocardial T1 30 min after manganese (ms)- Septal | 884±26 | 1030±48 | 919±31 | **<0.0001** | **<0.001** | 1029±49 | 918±28 | **<0.0001** | **<0.01** |
| Manganese influx (Ki; mL/100 g/tissue min)- Septal | 8.2±1.1 | 5.1±0.5 | 6.6±0.5 | **<0.0001** | **<0.0001** | 5.1±0.5 | 6.6±0.6 | **<0.0001** | **<0.001** |

*Controls v patients with takotsubo syndrome at index presentation
** Controls v patients with takotsubo syndrome at follow-up
† Controls v patients with takotsubo syndrome (focal pathology excluded) at index presentation
†† Controls v patients with takotsubo syndrome (focal pathology excluded) at follow-up

**Supplementary Table 6: Native T1, post-manganese T1 and myocardial manganese uptake (Ki) in all patients and in takotsubo syndrome excluding dual pathology.**

| | **Matched Control (n=20)** | **All takotsubo syndrome (n=20)** | | **P value*** | **P value**** | **Takotsubo excluding dual pathology (n=18)** | | **P value†** | **P value††** |
|---|---|---|---|---|---|---|---|---|---|
| | | Acute | Follow up | | | Acute | Follow up | | |
| Native T1 (ms)- Septal | 1211±28 | 1358±49 | 1238±35 | **<0.0001** | **0.02** | 1351±65 | 1230±28 | **<0.0001** | **0.03** |
| Myocardial T1 30 min after manganese (ms)- Septal | 884±26 | 1030±48 | 919±31 | **<0.0001** | **<0.001** | 1027±45 | 920±7 | **<0.0001** | **<0.01** |
| Manganese influx (Ki; mL/100 g/tissue min)- Septal | 8.2±1.1 | 5.1±0.5 | 6.6±0.5 | **<0.0001** | **<0.0001** | 5.0±0.5 | 6.7±0.4 | **<0.0001** | **<0.001** |

*Controls v patients with takotsubo syndrome at index presentation
** Controls v patients with takotsubo syndrome at follow-up
† Controls v patients with takotsubo syndrome (dual pathology excluded) at index presentation
†† Controls v patients with takotsubo syndrome (dual pathology excluded) at follow-up
